# Efficacy of Compounds Isolated from *Streptomyces olivaceus* against the Morphogenesis and Virulence of *Candida albicans*

**DOI:** 10.3390/md17080442

**Published:** 2019-07-26

**Authors:** Lili Meng, Changli Sun, Chunyan Zhang, Shihao Song, Xiuyun Sun, Jianhua Ju, Yinyue Deng

**Affiliations:** 1 School of Pharmaceutical Sciences (Shenzhen), Sun Yat-sen University, Guangzhou 510275, China; 2 College of Agriculture, South China Agricultural University, Guangzhou 510642, China; 3 CAS Key Laboratory of Tropical Marine Bio-Resources and Ecology, South China Sea Institute of Oceanology, Chinese Academy of Sciences, 164 West Xingang Road, Guangzhou 510301, China

**Keywords:** *Streptomyces olivaceus*, *Candida albicans*, hypha formation, virulence

## Abstract

*Candida albicans* is a type of commensal fungi which causes serious infections in immunocompromised patients and contributes to high mortality. In the present study, we identified that the extract from *Streptomyces olivaceus* SCSIO T05 inhibited hypha and biofilm formation of *C. albicans*. Seven compounds were isolated and evaluated for their effects on the biological functions and virulence of *C. albicans*. Two leading compounds, compound **1** (sorbicillin) and compound **2** (3-methyl-*N*-(2′-phenethyl)-butyrylamide) were identified as exhibiting strong activity against *C. albicans* morphological transition, adhesion activity, cytotoxicity, and adhesion to human cells, in a dose-dependent manner. Notably, compound **2** inhibited *C. albicans* infection in mouse oral mucosal models. Transcriptomic analysis and real-time PCR results revealed that compound **2** most likely inhibited the biological functions of *C. albicans* cells by regulating the expression levels of *HWP1*, *TEC1*, *ALS1*, *IFD6*, and *CSH1*, which are associated with filament formation and cell adhesion. Our results suggest that the candidate compounds present excellent efficacy against *C. albicans* pathogenicity and that they can be developed as potential options for the clinical treatment of candidiasis.

## 1. Introduction

The incidence of life-threatening infections caused by opportunistic fungi has increased sharply, and the most frequently encountered of these fungi is *Candida albicans*, accounting for 48% of the cases of bloodstream infections and contributing to a high mortality rate of 40% [1]. Unlike other commensal pathogens, *C. albicans* can survive in several distinct sites with different physiological pressures; therefore, various diseases may be caused when individuals are immunocompromised and debilitated [2,3].

There exist at least three morphologies for this fungus: the yeast budding form, pseudohyphae, and the filamentous form [4]. *C. albicans* cells begin infection with adhesion to host cells in the yeast budding form, followed by transitions from yeast to hypha [4,5,6,7]. These transitions are considered to be essential to the virulence of *C. albicans.* Additionally, infections caused by *C. albicans* are complex and are related to many other virulence factors such as adhesins, thigmotropism, and secretion of hydrolases [8]. Gradually, the hyphal form develops into spatially organized structures, namely biofilms, which are complex structures consisting of cells surrounded by exopolymeric matrices [9]. *Candida* biofilm formation has serious repercussions due to the notorious resistance of these biofilms to antifungal drugs and their negative impacts on implanted medical devices [10,11,12].

In recent decades, investigations have revealed that morphogenesis of *C. albicans* is mainly determined by environmental signals [13,14]. Furthermore, two well-characterized signaling pathways underlying the morphological changes are the cAMP-protein kinase A (PKA) pathway and the mitogen-activated protein kinase (MAPK) pathway. In these two pathways, *HWP1* is a type of cell wall protein that is critical for cellular adhesion and filamentous formation [13,15,16]; *EFG1* is a transcription factor that is activated by the PKA pathway and interacts directly with hypha-specific promoters during filament development [17,18]; and *TEC1*, which is regulated by *EFG1*, also induces filamentous formation [19]. The regulatory networks are also involved in cell adhesion. The *ALS* gene family, which encodes a large number of cell surface glycoproteins, plays an important role in *Candida* adherence to host cell surfaces [20,21].

The deep-sea strain *Streptomyces olivaceus* SCSIO T05 was isolated from a sediment sample collected from the Indian Ocean. Chemical investigation led to the isolation of anthracene scaffolds and xiamycins from the wild-type strain [22]. The mutant strain *S. olivaceus* SCSIO T05 ΔrsdK2/ΔxmcP was constructed for enhanced production of ansamycins [23]. Intriguingly, we found that the crude extract from the mutant strain exhibited strong inhibitory activity against the yeast-to-hypha transition and adhesion of *C. albicans*. In this study, seven compounds including sorbicillin (**1**), 3-methyl-*N*-(2′-phenylethyl) butyramide (**2**), 2-methyl-*N*-(2′-phenylethyl)-butyramide (**3**), cyclo-(l-Val-l-Pro) (**4**), cyclo-(l-Ile-l-Pro) (**5**), cyclo-(l-Leu-l-Pro) (**6**), and cyclo-(l-Phe-l-Pro) (**7**), were isolated and identified by comparing their MS and NMR data with those previously reported [24,25]. Among these compounds, sorbicillin (compound **1**) and 3-methyl-*N*-(2′-phenylethyl)-butyramide (compound **2**) exhibited excellent efficacy against yeast-to-hypha transition and adhesion of *C. albicans*. Additionally, these two compounds inhibited the virulence of *C. albicans* toward a human cell line, and compound **2** showed good inhibitory activity in mouse infection models. In general, our findings suggest that the two compounds are potential novel antifungal agents against mucosal candidiasis.

## 2. Results

### 2.1. Extract of S. olivaceus SCSIO T05 ΔrsdK2/ΔxmcP Inhibits Hypha Formation and Adhesion of C. albicans

**Figure 1:**
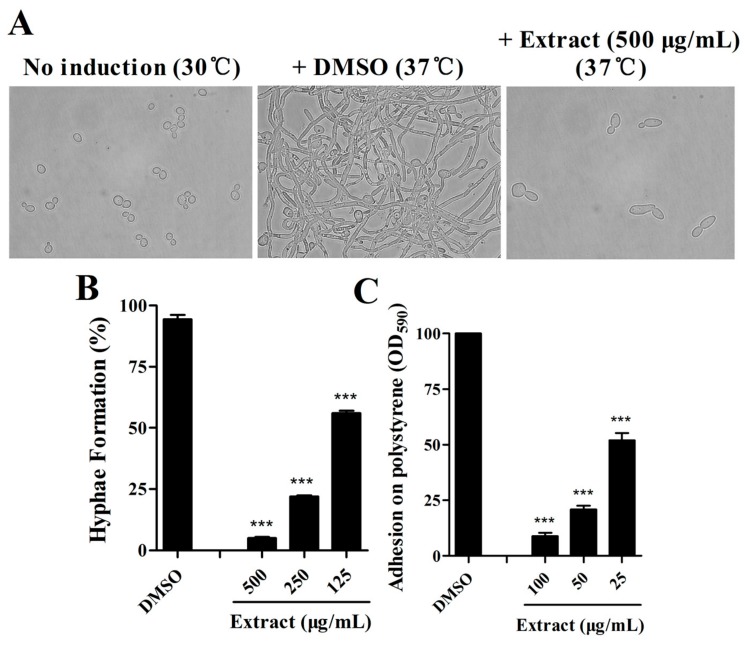
Effects of the extract from *Streptomyces olivaceus* SCSIO T05 ΔrsdK2/ΔxmcP on hypha formation in *Candida albicans*. *Candida* cells were grown under noninductive conditions (30 °C) or with induction (37 °C). The photographs were taken 6 h after induction (**A**), and hypha formation rates were calculated (**B**). Efficacy of the extract against adhesion of *C. albicans* at 100 μg/mL, 50 μg/mL and 25 μg/mL, respectively (**C**). The data represent the means ± standard deviations of three independent experiments. ***, *p* < 0.001 (unpaired *t*-test).

For *C. albicans*, morphogenesis of the yeast–to–hypha transition is considered a key virulence factor, and adhesion is the first step for biofilm formation. To determine whether there are some active compounds from *S. olivaceus* that exhibit efficacy against *C. albicans*, we obtained crude extracts of compounds from *S. olivaceus* SCSIO T05 rsdK2/xmcP and tested the effects of the extracts on morphology and adhesion in *C. albicans*. The crude extract decreased filament formation in *C. albicans* by 94.96 ± 0.82% and 75.16 ± 0.82% when added at final concentrations of 500 μg/mL and 250 μg/mL, respectively (Figure 1A,B). Intriguingly, the extract also inhibited *Candida* adhesion activity to 13.07 ± 1.75%, 24.95 ± 1.86%, and 58.52 ± 4.64% of that observed in the untreated control when added at lower final concentrations of 100 μg/mL, 50 μg/mL, and 25 μg/mL, respectively (Figure 1C).

### 2.2. Isolation of Active Compounds from the Extract

**Figure 2:**
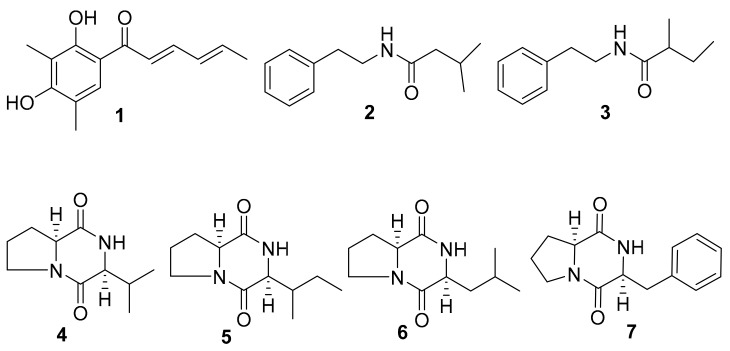
Structures of compounds **1**–**7** isolated from the extract of *S. olivaceus*.

**Figure 3:**
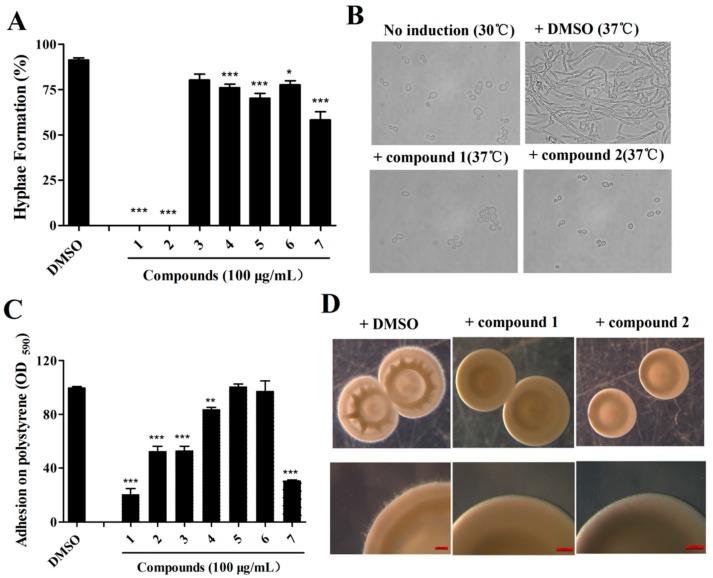
Effects of compounds isolated from the extract of *S. olivaceus* on hypha formation and adhesion of *C. albicans*. (**A**) Effects of isolated compounds on the hyphal formation of *C. albicans* SC5314 at 100 μg/mL. (**B**) Photograph of *C. albicans* SC5314 cells, which were grown under noninductive conditions (30 °C), or with induction (37 °C), with or without compounds **1** or **2** at 100 μg/mL. (**C**) Effects of isolated compounds on adhesion assays of *C. albicans* SC5314 at 100 μg/mL. (**D**) Colony morphology of *C. albicans* treated with compounds **1** and **2** at 100 μg/mL on Spider agar plates with DMSO as a control. The data represent the means ± standard deviations of three independent experiments. *, *p* < 0.05; **, *p* < 0.01; ***, *p* < 0.001 (unpaired *t*-test).

All isolated compounds (**1**–**7**, Figure 2 and Table S1) were evaluated for antifungal activity. Among these, compounds **1** and **2** exhibited excellent efficacy in the inhibition of the yeast-to-hypha transition of *C. albicans* and decreased the hyphal formation rates to 0% at 100 μg/mL (Figure 3A–C). In the adhesion assays, compounds **1**–**3** and compound **7** exhibited strong inhibition against *C. albicans* at 100 μg/mL (Figure 3C). We investigated whether the two compounds affected the colony morphology of *C. albicans*. As shown in Figure 3D, similar to the effects on filament formation, the two compounds also clearly altered the colony morphology of *C. albicans*. There were numerous hyphae around the margins of the *C. albicans* cell colonies that disappeared in the presence of the two compounds (Figure 3D). Moreover, the addition of compounds **1** or **2** also altered the colonies of *C. albicans* from wrinkled to smooth (Figure 3D).

### 2.3. Effects of Isolated Compounds on the Cytotoxicity of C. albicans

**Figure 4:**
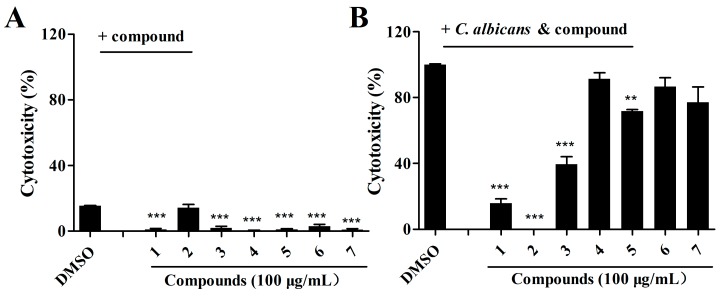
Effects of the isolated compounds on *C. albicans* virulence using the cell line A549. (**A**) Analysis of the toxicity of compounds toward A549 cells. The compounds were dissolved in DMSO, and the amount of DMSO used as the solvent for the compounds was used as a control. (**B**) Analysis of the effects of the compounds on the cytotoxicity of *C. albicans* toward A549 cells. Cytotoxicity was detected and measured as lactate dehydrogenase (LDH) release. The LDH released by A549 cells after inoculation with *C. albicans* in the absence of compounds was defined as 100% to normalize the LDH release ratios of the other treatments. The data represent the means ± standard deviations of three independent experiments. **, *p* < 0.01; ***, *p* < 0.001 (unpaired *t*-test).

Cytotoxicity was measured by quantifying the release of lactate dehydrogenase (LDH) into the supernatants of a human cell line, A549. To assess the effects of candidate compounds on the pathogenicity of *C. albicans*, we then continued to investigate whether these compounds affected *C. albicans* cytotoxicity to A549 cells. The results shown in Figure 4A suggest that compounds **1**, **2**, and **3** (100 μg/mL) did not exhibit toxic effects on A549 cells at a concentration of 100 μg/mL. However, these three compounds were capable of attenuating the cytotoxicity of *C. albicans*, and exogenous addition of the same concentration of these compounds reduced *C. albicans* virulence by 79.09 ± 3.53%, 100.00%, and 58.44 ± 5.45%, respectively (Figure 4B).

### 2.4. The Effects of Compound ***1*** and ***2*** on C. albicans Are Dose-Dependent

**Figure 5:**
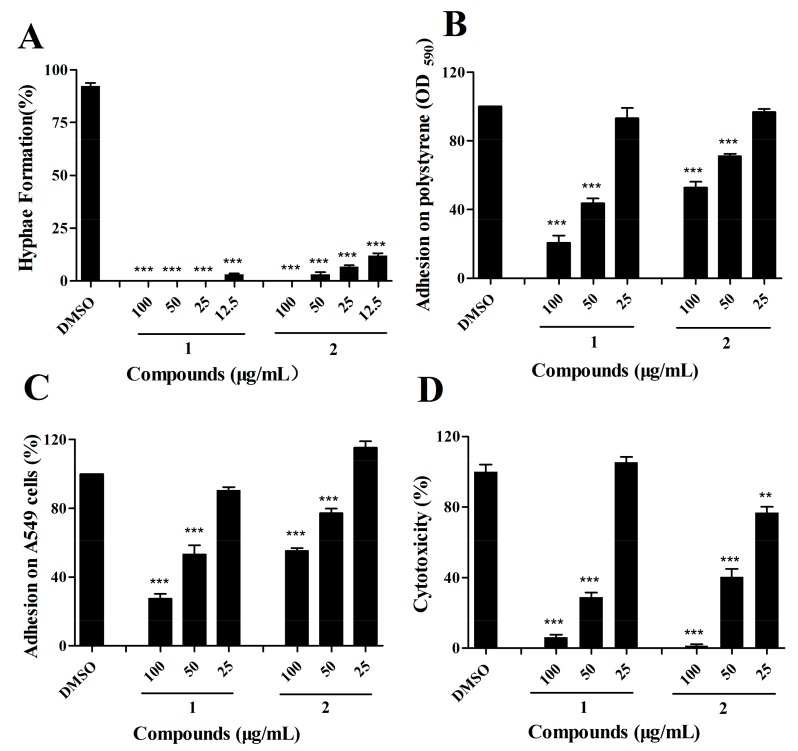
Effects of the candidate compounds on hypha formation (**A**), adhesion on polystyrene (**B**), adhesion on epithelial cell A549 (**C**), and cytotoxicity (**D**) assays at gradient concentrations of 100 μg/mL, 50 μg/mL, and 25 μg/mL. The data represent the means ± standard deviations of three independent experiments. **, *p* < 0.01; ***, *p* < 0.001 (unpaired *t*-test).

Due to the excellent activity of compounds **1** and **2** in the reduction of the yeast-to-hypha transition, adhesion, and cytotoxicity of *C. albicans* SC5314 cells, we then investigated whether these two compounds affected *C. albicans* in a dose-dependent manner. Gradient concentrations of compounds **1** and **2** were assessed for inhibitory activity against *C. albicans*. The two compounds exhibited dose-dependent effects on the hypha formation, adhesion assays, and cytotoxicity (Figure 5A–D). In particular, compounds **1** and **2** inhibited the virulence of *C. albicans* by 67.25 ± 3.84% and 53.49 ± 5.71%, respectively, at a final concentration of 50 μg/mL (Figure 5D). Analysis of the growth rates indicated that compound **2** hardly influenced the growth of *C. albicans* at 100 μg/mL, while compound **1** partially inhibited this fungus growth at a final concentration of 50 μg/mL (Figure S2).

### 2.5. Compound ***2*** Reduced C. albicans Infection in the Mouse Oral Mucosal Infection Model

**Figure 6:**
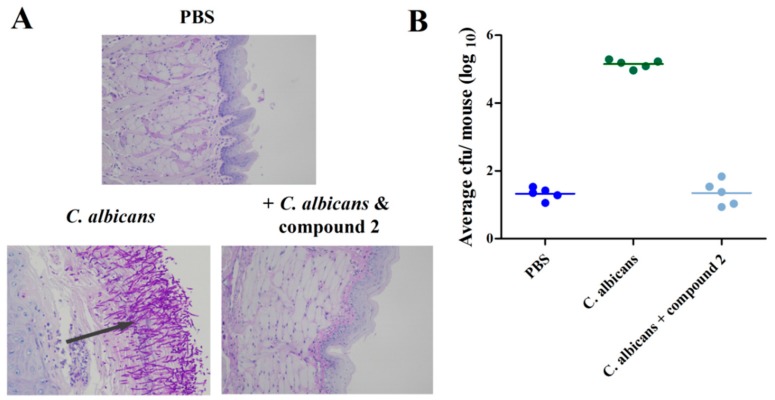
Efficacy of compound **2** (100 μg/mL) against *C. albicans* SC5314 in the mouse oral mucosal infection model. Pathological sections were evaluated to determine the effect on *C. albicans* infection (**A**). The in vivo pathogen cell numbers of *C. albicans* SC5314 in the mouse tongues after infection in the absence and presence of compound **2** (**B**).

Due to the fact that compound **2** did not inhibit the growth of *C. albicans* cells, we continued to investigate the effects of compound **2** in vivo using a mouse oral mucosal infection model. Treatment with compound **2** significantly inhibited the number of *C. albicans* cells aggregated in the pathological tissues compared to tissues infected with only fungus (Figure 6A,B). Measurement of the fungus colony-forming units (CFUs) in the mouse tongues revealed that the addition of 100 μg/mL of compound **2** decreased the CFU count from 1.4 × 10^5^ to 22 at five days post-inoculation (Figure 6B).

### 2.6. Compound ***2*** Affected the Expression Levels of a Wide Range of Genes in C. albicans

**Figure 7:**
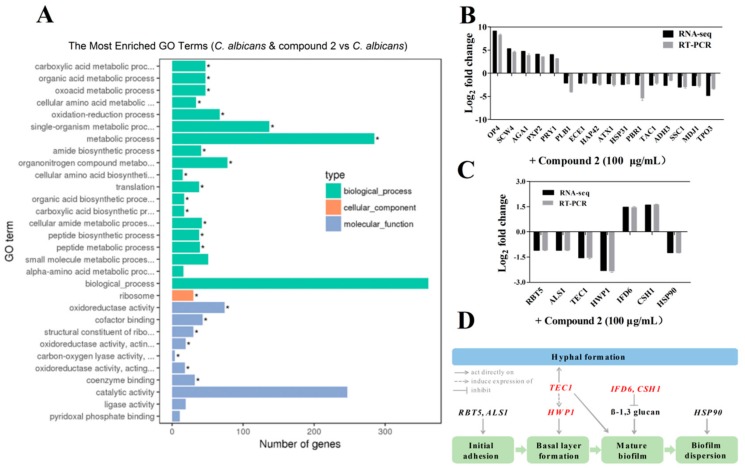
Differential gene expression profiles of *C. albicans* treated with compound **2** (100 μg/mL). GO term enrichment analysis of gene expression levels measured by RNA-seq (**A**). qRT-PCR analysis of the selected genes confirmed the RNA-seq results (**B**). Effect of compound **2** on the expression levels of genes associated with morphogenesis, adhesion and biofilm formation (**C**). Schematic diagram of the signaling pathways that govern each phase of biofilm formation and morphogenesis in *C. albicans* affected by compound **2** (**D**). The data represent the means ± standard deviations of three independent experiments. *, the enriched GO term.

To determine the effect of compound **2** on the gene expression profile of *C. albicans*, we analyzed and compared the transcriptome profiles of the *C. albicans* wild-type SC5314 strain cultured in the presence and absence of compound **2** using RNA-seq. Differential gene expression analysis identified 220 genes with a log_2_ fold change ≥1.5 in the wild-type strain SC5314 upon the addition of 100 μg/mL of compound **2** (Figure 7A and Table S2). These differentially expressed genes are related to a wide range of biological functions such as transcriptional regulation, membrane components, signal transduction, motility, flagellum synthesis, stress tolerance, metabolism, and virulence (Figure 7A and Table S2). Quantitative RT-PCR analysis of the selected genes confirmed the RNA-seq results (Figure 7B,C). Intriguingly, GO term analysis indicated that many of these genes are involved in hypha formation and biofilm formation, such as the downregulated genes *HWP1*, *TEC1*, and *ALS1*, which have been well studied in *C. albicans* based on previous investigations (Figure 7C,D). In addition, our results suggested that the candidate compound may inhibit the pathogenesis of *C. albicans* via a complex signaling network (Figure 7C,D).

## 3. Discussion

*C. albicans* is the most common opportunistic human pathogen and may cause fatal infections in patients with immune deficiency [26]. Unlike other fungal pathogens that infect only some specific sites, *C. albicans* can survive as a commensal in several anatomically different sites, can switch between yeast and hypha forms in response to environmental changes [2,26], and gradually forms biofilm. Morphogenesis is regarded as a key virulence factor, and biofilms are intrinsically resistant to antifungal drugs such as amphotericin B, azoles, and echinocandin, while adhesion is the inceptive phase of mature biofilm formation [27]. As the development of new drugs using traditional target-based methods is associated with low productivity, there has been increased interest in new therapeutics and strategies to treat fungal diseases [27,28]. Therefore, overcoming the increasing drug resistance of *Candida* is a challenge worldwide.

As most antibiotics are produced by actinomycetes, we focused on the investigation of the antifungal activity of metabolites of *S. olivaceus*. The aim of this study was to estimate the anti-*Candida* activity of the seven compounds isolated from *S. olivaceus* SCSIO T05 ΔrsdK2/ΔxmcP because the extract of this strain exhibited excellent inhibition of yeast–to–hypha transition and adhesion at regular screening concentrations (Figure 1; Figure 2). Compounds **1** and **2** (100 μg/mL) exhibited strong inhibition of morphological transition, adhesion, and cytotoxicity in a dosage-dependent manner (Figure 3; Figure 4; Figure 5). A prior study identified that sorbicillin (compound **1**) can be isolated from *Penicillium* spp. and has good bioactivity and wide pharmaceutical applications [29]; however, the characteristics of compound **2** are not clear. Nowadays, sharply increasing drug resistance is a serious problem. To treat the infections caused by microorganisms, people take antibiotics which usually kill microbes or inhibit their growth. In this way, antibiotics induce enormous environmental pressure. Previous investigations have asserted that drug resistance is natural selection in that microorganisms able to avoid the pressure will be the ones to propagate with resistance genes and gradually, drug resistance phenomena emerge [30]. Given the rapid spread of drug resistance [30,31], agents that are virulence inhibitors rather than fungicides are urgently required; that is, agents which do not inhibit the growth of pathogen cells. Our findings indicated that compound **2** showed almost no inhibition of the growth of *C. albicans* at 100 μg/mL (Figure S2) but displayed excellent inhibitory activity against *C. albicans* in vitro (100 μg/mL) and effectively prevented *Candida* infection in mouse oral tissue. In addition, the transcriptomic profile results of *C. albicans* in the absence and presence of compound **2** revealed that compound **2** interfered with the expression of some important proteins, namely Hwp1, TEC1, IFD6, and CSH1, which are associated with hypha formation and biofilm formation [20,32,33,34,35,36,37]. These results suggested that compound **2** might be a good candidate for the development of new antivirulence and antibiofilm agents against *C. albicans* diseases. 

Interestingly, compound **2** was reported as an inhibitor of quorum sensing (QS)-controlled phenotypes in the gram-negative bacterium *Vibrio harveyi*, and this characteristic may be attributed to the effect of compound **2** on the QS systems of this pathogen [38]. As the phenotypes affected by compound **2** in *C. albicans* were linked to QS signals produced by *C. albicans* cells [13], compound **2** may have some influence on QS in *C. albicans*, and this aspect needs further investigation. Furthermore, the simple structure of compound **2** makes it very easy to produce by chemical synthesis. This compound can be obtained by a one-step chemical reaction of 3-methyl-butyric acid with phenylethylamine. The biosynthetic gene cluster and biosynthetic pathway of compound **2** can be assigned by bioinformatics analysis as the genome sequence of *S. olivaceus* has been identified [22,23]. The application of molecular biological, biochemical, and microbiological tools can also enhance the production of this compound. Therefore, we hypothesize that compound **2** may be a potentially promising antifungal drug. However, more work will be performed in the future to overcome some defects in our study; for example, to test the efficacy of compound **2** on the systemic infection model, and for the mouse mucosal model, infection followed by compound treatment will be more convincing.

## 4. Materials and Methods

### 4.1. General Experimental Procedures

NMR spectra were acquired with an AVANCE 500 spectrometer (Bruker, Zurich, Switzerland) or AVANCE HD 700 spectrometer (Bruker, Zurich, Switzerland) with TMS as the internal standard. Chemical shifts (δ) were expressed in ppm with reference to the solvent signals. Mass spectral data were obtained on a MaXis quadrupole-time-of-flight mass spectrometer (Bruker, Zurich, Switzerland). Silica gel (100–200 mesh, Yantai Jiangyou Silica Gel Development Co., Ltd., Yantai, China) and an RP-18 column (40–63 μm, Millipore, Massachusetts, USA) were applied for column chromatography (CC). An Agilent 1260 liquid chromatograph (Agilent Technologies Inc., California, USA) with a diode array detector (DAD) and a YMC-Pack ODS-A column (250 × 20 mm, 5 μm) was used for semipreparative HPLC.

### 4.2. Strains and Reagents

*C. albicans* SC5314 (ATCC^®^ MYA-2876TM) was grown and maintained in YPD medium (1% yeast extract, 2% peptone, 2% glucose) at 30 °C with shaking at 200 rpm (4 g). The compounds were dissolved in dimethyl sulfoxide (DMSO, Sigma, Shanghai, China) (10 mg/mL) and stored at 4 °C. The wild-type strain of SCSIO T05 was isolated from a sediment sample collected from the Indian Ocean at E94°3364′ and S1°4256′ at a depth of 4617 m, which was identified as *S. olivaceus* due to its 16S rRNA gene sequence analyses and comparisons with other established sequences. The 16S rDNA gene sequence has been deposited with GenBank (accession number MF429815). The mutant strain *S. olivaceus* SCSIO T05 ΔrsdK2/ΔxmcP was constructed by gene disruption [23].

### 4.3. Fermentation and Isolation

The fermentation procedure of the mutant strain was as described previously [23]. The entire culture broth (40 L) was harvested and filtered to yield the mycelium cake and liquid broth. The mycelium cake was extracted by ultrasonication with acetone, and the liquid broth was extracted with butanone. The acetone layer and butanone layer were evaporated to dryness to yield two residues, respectively. The two residues were combined after HPLC analysis and subjected to silica gel CC using gradient elution with CHCl_3_ and MeOH mixtures (100:0, 95:5, 90:10, 85:15, 80:20, 70:30, 50:50) to obtain seven fractions (Fr.1–Fr.7). Fr.2 was subjected to Rp-18 CC and eluted with MeCN-H_2_O (30:70→100:0, 0→120 min) to afford five fractions (Fr.2-1–Fr.2-5). Compound **1** (5.0 mg) was yielded by semipreparative HPLC (MeCN-H_2_O, 5:5→10:0, 0→30 min) from Fr.2-1; Fr.3 was separated by Rp-18 CC with MeCN-H_2_O (5:95→100:0, 0→120 min) to afford five fractions (Fr.3-1–Fr.3-5). Fr.3-2 was subjected to semipreparative HPLC (MeCN-H_2_O, 10:90) to yield compound **3** (1.9 mg). Compounds **5**–**7** (7.3 mg, 11.2 mg, 8.0 mg, respectively) were obtained by semipreparative HPLC (MeCN-H_2_O, 15:85, 18:82) from Fr.3-3 and Fr.3-4; Fr.4 was fractionated by Rp-18 CC and eluted with MeCN-H_2_O (5:95→100:0, 0→120 min) to afford four fractions (Fr.4-1–Fr.4-4), and Fr.4-2 was separated by semipreparative HPLC (MeCN-H_2_O, 15:85) to obtain compound **4** (5.4 mg); Fr.5 was subjected to Rp-18 CC and eluted with MeCN-H_2_O (5:95→100:0, 0→120 min) to afford Fr.5-1–Fr.5-6. Compound **2** (8.5 mg) was yielded by semipreparative HPLC (MeCN-H_2_O, 30:70) from Fr.5-2.

### 4.4. Adhesion Assays

With modifications of the previous published protocol [5,39], adhesion of *C. albicans* on polystyrene was examined in the presence or absence of compounds, with DMSO as a negative control and cell-free wells as a blank control. Overnight culture cells were diluted to an optical density (OD_600_) of 0.5 using fresh glucose minimal medium (GMM; 6.7 g Bacto yeast nitrogen base and 0.2% glucose per liter). A 200-μL aliquot of cell suspension with compound (100 μg/mL) was added into a 96-well plate and incubated at 37 °C for 4 h. *Candida* cells were dyed with 0.5% crystal violet for 45 min and then washed with cold water to remove the dyestuff and planktonic cells. Each well was filled with 200 μL of 75% ethyl alcohol and was measured for absorbance of the dye at 590 nm.

### 4.5. Cell Binding Assays

To estimate the efficacy of compounds in the inhibition of adhesion on the epithelial cells of yeast cells, which was considered the first step of invasion, the human cell line A549 was employed for the measurement of adhesion ability, following the methods in the previous publications with small modifications [5,40]. The A549 cells (5 × 10^3^ per well) were grown overnight in Dulbecco’s modified Eagle’s medium (DMEM) containing 10% fetal bovine serum (FBS) on a 96-well tissue culture plate, then were washed with PBS (phosphate buffered saline) twice. Compound preparation and suspension was performed using the same method as described for the biofilm formation assay, using DMSO and GMM as controls. These 96-well plates were incubated at 37 °C for 1.5 h, followed by the same treatment as mentioned in the adhesion assays. Finally, cell binding on A549 cells was quantified by measuring the absorbance at 590 nm.

### 4.6. Hypha Formation Assays

An overnight culture was diluted to an OD_600_ of 0.1 using fresh GMM [5]. After treatment with compounds (100 μg/mL) or DMSO, the cultures were cultivated at 37 °C for 6 h, the yeast cells were harvested by centrifugation at 5000 rpm (2348× *g*) for 10 min and then observed directly under a Leica inverted fluorescence microscope (Leica, Wetzlar, Germany).

### 4.7. Colony Morphology Assays

An overnight culture was diluted to 2 × 10^3^ colony-forming units (CFUs)/mL and incubated on Spider medium agar plates (1% peptone, 1% mannitol, 0.2% K_2_HPO_4_, and 1.5% agar) containing compounds (100 μg/mL) or DMSO at 37 °C for 24 h [5]. These plates were visualized directly under a Leica DMi8 microscope (Leica, Wetzlar, Germany), and images were captured using a Nikon Coolpix digital camera (Nikon Corporation, Tokyo, Japan).

### 4.8. Cytotoxicity Assays

The A549 cells were cultured with the same method as in the adhesion assays (1.5 × 10^4^ cells per well) [39]. Overnight cultures of *C. albicans* were diluted to an OD_600_ of 0.1 in DMEM containing 1% FBS, with or without the addition of compounds. By following the manufacturer’s instructions for the cytoTox 96^®^ non-radioactive cytotoxicity assay kit (Promega, Madison, WI, USA), cytotoxicity was determined by measuring the release of lactate dehydrogenase (LDH) into the supernatants at 490 nm. The cytotoxicity of the compounds was tested using the same method.

### 4.9. Cell Growth Analysis

For this assay, *C. albicans* cells were incubated overnight and then freshly diluted to an OD_600_ of 0.05 using GMM. Then, 300-μL aliquots of cell suspension containing compounds (100 μg/mL) were added to the plate in triplicate. Growth analysis was then performed on the Bioscreen-C automated growth analysis system (Oy Growth Curves Ab Ltd, Helsingfors, Finland) at 30 °C for 2 days.

### 4.10. Quantitative Real-Time PCR Analysis

*C. albicans* cells were grown in GMM at 30 °C and then diluted in the same medium to an OD_600_ of 0.1 with the tested compounds (100 µg/mL), using DMSO as control. After incubation for 6 h at 37 °C, cells switched from yeast to hyphal form and then cell samples were collected and washed with PBS. Total RNA was extracted using an RNA extraction kit (Promega, Beijing, China) and quantified. cDNA was obtained via a reverse transcription reaction using a reverse transcription kit (TaKaRa Biotechnology, Dalian, China) with the primers shown in Table S3. In this experiment, 4 μL of cDNA (10 ng/μL), 0.5 μL forward primer, 0.5 μL reverse primer, and 5 μL SYBR qPCR master mix (Vazyme, Nanjing, China) were mixed for each well. Real-time PCR analysis was performed on a 7300Plus real-time PCR system (Applied Biosystems, Massachusetts, USA). GSP1, a housekeeping gene in this fungus, was used as the standard for other gene expression [41]. The target gene expression results were calculated using the comparative CT (ΔΔCT) method. We performed this experiment in triplicate with four repeats for each sample. As for RNA-sequencing, we routinely extracted the RNA samples mentioned above and then RNA-seq analysis was carried out by the Novogene company (Beijing, China). We obtained our results based on the Illumina technique and Paired-end method [42].

### 4.11. Mouse Oral Mucosal Assays

The establishment of a mouse oral mucosal model was based on a published study with minor modifications [43,44]. In this experiment, 20–22 g male BaLB/c mice (3 mice per group; animal experiment permission number 2019-C004) were subcutaneously injected with hydrocortisone (225 mg/kg) dissolved in PBS containing 0.5% Tween-20 on the first day. Then, on the following day, *Candida* cells were washed with Hank’s balanced salt solution (Biohao Biotechnology Co., Ltd., Wuhan, China) and then twice with PBS. The cells were resuspended in PBS (OD_600_ = 0.1, equal to 10^6^ CFU/mL) in the absence or presence of candidate compounds at a final concentration of 100 μg/mL. After intraperitoneal injection of about 70–80 μL 0.1 g/mL chloral hydrate (Yuanye Biotech, Shanghai, China), the anesthetized mice were placed on an isothermal mat maintained at 37 °C, and cotton balls soaked with pathogenic cells were placed under the tongues of the mice for 75 min. On the fifth day, tongues of sacrificed mice were dissected for further analysis using pathological sections and CFU analysis. Analysis of CFUs was performed on SDA agar medium (Sabouraud agar plate medium; 40 g maltose, 10 g peptone, and 20 g agar per liter, pH 6.2). After homogenizing the tongue tissue, an aliquot of 200 μL was spread on the SDA plates, and CFUs were determined after 24 h.

## Supplementary Materials

The following are available online at https://www.mdpi.com/1660-3397/17/8/442/s1. Figure S1: ESIMS spectrum of compounds 1 and 2; Figure S2: Growth curves of *C. albicans* cells treated with compound 1 (50 μg/mL) and compound 2 (100 μg/mL); Table S1: List of compounds used in this study; Table S2: List of genes differentially expressed in *C. albicans* treated with compound 2 (Log_2_ fold change ≥1.5); Table S3: PCR primers used in this study.
