# Supplementary material for: Efficacy of Compounds Isolated from Streptomyces olivaceus against the Morphogenesis and Virulence of Candida albicans
Source: Mar Drugs. 2019 Jul 26;17(8):442. doi: 10.3390/md17080442 (PMC6723460; doi:10.3390/md17080442)

Supplementary Information

Efficacy of compounds isolated from *Streptomyces olivaceus* on the morphogenesis and virulence of *Candida albicans*

Lili Meng[1,2#], Changli Sun[3#], Chunyan Zhang[3], Shihao Song[1,2], Xiuyun Sun[1,2], Jianhua Ju[3], Yinyue Deng[1*]

[1]*School of Pharmaceutical Sciences (Shenzhen), Sun Yat-sen University, Guangzhou 510275, China*

[2]*College of Agriculture, South China Agricultural University, Guangzhou 510642, China*

[3]*CAS Key Laboratory of Tropical Marine Bio-resources and Ecology, South China Sea Institute of Oceanology, Chinese Academy of Sciences, 164 West Xingang Road, Guangzhou 510301, China*

[#]These authors contributed equally to this work.

***Corresponding author:** Yinyue Deng: yinyue_deng@hotmail.com

Tel.:(+86)2085286471; Fax: (+86)2085280203

Table S1. List of compounds used in this study (The leading compounds were highlighted in red color).

Structure & name	^{1}H-NMR descriptions	^{13}C-NMR descriptions
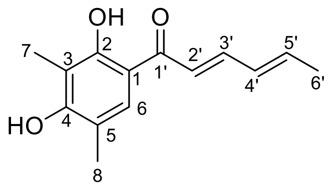 Sorbicillin (**1**)	7.44 (1H, s, H-6), 2.22 (3H, s, H-8), 2.15 (3H, s, H-7), 1.91(3H, d, *J* = 6.4 Hz, H-6′).	110.4 (s, C-1), 162.6 (s, C-2), 113.5 (s, C-3), 158.7 (s, C-4), 114.5 (s, C-5), 128.8 (d, C-6), 7.5 (q, C-7), 15.6 (q, C-8), 192.5 (s, C-1′), 121.9 (d, C-2′), 144.5 (d, C-3′), 130.6 (d, C-4′), 141.3 (d, C-5′), 18.9 (q, C-6′)
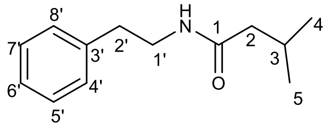 3-methyl-N-(2'-phenylethyl) butyramide (**2**)	7.16-7.27 (5H, m, H-4′, 5′, 6′, 7′, 8′), 3.39(2H, m, H-1′), 3.40 (2H, t, *J* = 7.3 Hz, H-2), 2.78 (2H, t, *J* = 7.5 Hz, H-2′), 2.01 (1H, overlapped, H-3′), 2.01 (2H, overlapped, H-1′), 0.89 (6H, d, *J* = 6.7 Hz, H-4 and H-5)	174.2 (s, C-1), 40.5(t, C-2), 26.0 (d, C-3), 21.3(q, C-4 and C-5), 45.0 (t, C-1′), 35.2 (t, C-2′), 139.1 (s, C-3′), 128.4 (d, C-4′,8′), 128.1 (d, C-5′,7′), 125.9 (d, C-6′)
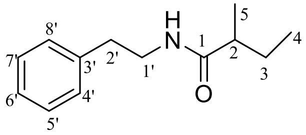 2-methyl-N-(2′-phenylethyl)-butyramide (**3**)	7.18-7.27 (5H, m, H-4′,5′,6′,7′,8′), 3.39(2H, m, H-1′), 2.80 (2H, m, H-2′), 2.14 (1H, m, H-2), 1.38 (2H, m, H-3), 1.04 (3H, d, *J* = 6.9 Hz, H-5), 0.83(3H, t, *J* = 7.4 Hz, H-4)	178.1(s, C-1), 42.2(d, C-2), 26.9 (t, C-3), 16..7(q, C-4), 10.9 (q, C-5), 40.4 (t, C-1′), 35.2 (t, C-2′), 139.2 (s, C-3′), 128.0 (d, C-4′,8′), 128.5 (d, C-5′,7′), 139.2 (d, C-6′)

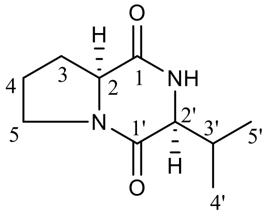 cyclo-(L-Val-L-Pro) (**4**)	4.20 (1H, m, H-2), 3.55 (1H, m, H-5a), 3.50 (1H, m, H-5b), 2.48 (1H, m, H-3a), 2.32 (1H, m, H-3b), 2.06 (1H, m, H-3′), 2.02 (1H, m, H-4a), 1.94 (1H, m, H-4b), 1.09 (3H, d, *J* = 7.2 Hz, H-4′), 0.93 (3H, d, *J* = 6.9 Hz, H-5′)	171.2 (s, C-1), 58.6 (d, C-2), 21.9 (t, C-3), 28.1 (t, C-4), 44.8 (t, C-5), 166.2 (s, C-1′), 60.1 (d, C-2′), 28.5 (d, C-3′), 17.5 (q, C-4′), 15.3 (q, C-5′)
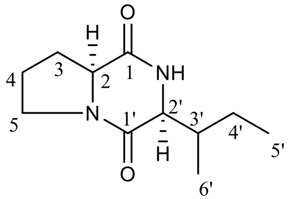 cyclo-(L-Ile-L-Pro) (**5**)	4.19 (1H, m, H-2), 4.07 (1H, br. s, H-2′), 3.63 (1H, m, H-5a), 3.56 (1H, m, H-5b), 2.32 (1H, m, H-3a), 2.16 (1H, m, H-3b), 2.03 (1H, m, H-4a), 1.93 (1H, m, H-4b), 1.44-1.31 (2H, m, H-4′), 1.06 (3H, d, J = 7.1 Hz, H-5′), 0.93 (3H, t, *J* = 7.4 Hz, H-5’)	166.2 (s, C-1), 58.6 (d, C-2), 21.8 (t, C-3), 28.1 (t, C-4), 44.8 (t, C-5), 171.0 (s, C-1′), 59.9 (d, C-2′), 35.7 (d, C-3′), 24.0 (t, C-4′), 11.1 (q, C-5′), 14.1 (q, C-6′)
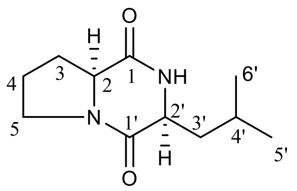 cyclo-(L-Leu-L-Pro) (**6**)	4.26 (1H, m, H-2), 4.12 (1H, br. s，H-2′)，3.64 (1H, m, H-5a), 3.51 (1H, m, H-5b), 2.30 (1H, m, H-3a), 2.01 (1H, m, H-4a), 1.91 (1H, m, H-4b), 0.96 (3H, d, *J* = 6.3 Hz, H-5′), 0.95 (3H, t, *J* = 6.3 Hz, H-6′)	167.5 (s, C-1), 58.9 (d, C-2), 21.9 (t, C-3), 27.7 (t, C-4), 45.0 (t, C-5), 171.4 (s, C-1′), 53.2 (d, C-2′), 38.0 (t, C-3′), 24.4 (d, C-4′), 21.9 (q, C-5′), 20.8 (q, C-6′)
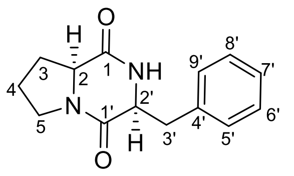 cyclo-(L-Phe-L-Pro) (**7**)	7.21- 7.29 (5H, m, H-5′, 6′, 7′, 8′, 9′), 4.44 (2H, t，*J* = 4.6 Hz, H-2′)，4.06 (1H, m, H-2), 2.08 (1H, m, H-4a), 1.80 (1H, m, H-4b)	169.5 (s, C-1), 58.7 (d, C-2), 21.4 (t, C-3), 28.0 (t, C-4), 44.7 (t, C-5), 165.5 (s, C-1′), 56.3(d, C-2′), 36.8(t, C-3′), 136.0 (s, C-4′), 128.1 (d, C-5′,9′), 129.7 (q, C-6′,8′), 126.7 (d, C-7′)

Table S2. List of genes differentially expressed (Log$_2$ fold change) in *C. albicans* treated by compound 2 (Log$_2$ fold change ≥ 1.5) .

Gene ID	Gene name[a]	Description	q-PCR	RNA-seq
CAALFM_C405580CA	-	hypothetical protein		9.92
CAALFM_C113080WA	OP4	Ala- Leu- and Ser-rich protein	8.28±0.24	9.41
CAALFM_C105890WA	-	hypothetical protein		7.40
CAALFM_C300920WA	ATO1	Putative fungal-specific transmembrane protein		6.22
CAALFM_C306580WA	JEN1	Lactate transporter		6.02
CAALFM_C202230CA	-	hypothetical protein		5.44
CAALFM_C102520WA	SCW4	Putative cell wall protein	4.63±0.15	5.32
CAALFM_C500540CA	AGA1	Protein with some similarity to agglutinin subunit	3.94±0.86	4.73
CAALFM_CR10320WA	-	hypothetical protein		4.64
CAALFM_C206680WA	FRP3	putative ammonium permease		4.27
CAALFM_C203350WA	PGA17	Putative GPI-anchored protein	4.48±0.87	4.20
CAALFM_C401070WA	HGT17	Putative MFS family glucose transporter		4.17
CAALFM_C301930WA	PXP2	Putative acyl-CoA oxidase	3.56±0.08	4.14
CAALFM_C107580CA	PRY1	Pry family pathogenesis-related protein	3.14±0.11	4.02
CAALFM_C202060CA	FMO1	Putative oxidoreductase		3.59
CAALFM_C404020CA	-	hypothetical protein		3.36
CAALFM_C108330CA	ADH2	alcohol dehydrogenase		3.23
CAALFM_C300930WA	ATO2	Putative fungal-specific transmembrane protein		3.16
CAALFM_C202970CA	ALD5	aldehyde dehydrogenase (NAD(P)(+))		3.11
CAALFM_CR02490WA	OPT4	Oligopeptide transporter		3.07
CAALFM_C401100CA	AGP2	Amino acid permease		3.07
CAALFM_C504380CA	-	hypothetical protein		3.04
CAALFM_C210350CA	ACS1	acetate--CoA ligase 1		3.03

CAALFM_C303580CA	GTT13	Putative glutathione S-transferase		2.91
CAALFM_C114020WA	-	hypothetical protein		2.85
CAALFM_C105960WA	PGA45	Putative GPI-anchored cell wall protein		2.78
CAALFM_C201020WA	HGT6	hexose transporter		2.60
CAALFM_C704180WA	FAA2-3	Predicted acyl CoA synthetase		2.59
CAALFM_C700290CA	HGT13	Predicted sugar transporter, involved in glycerol utilization		2.56
CAALFM_C301960CA	POX1-3	acyl-CoA oxidase		2.49
CAALFM_C101240WA	IFD3	Putative aldo/keto reductase		2.47
Novel00031	-//-			2.43
CAALFM_C205250CA	AAT1	aspartate transaminase		2.41
CAALFM_C205820WA	-	hypothetical protein		2.40
CAALFM_C106810WA	CAT1	catalase A		2.29
CAALFM_CR08990CA	-	hypothetical protein		2.25
CAALFM_C604620CA	NAG4	Putative transporter; fungal-specific		2.24
CAALFM_C307160WA	PGA32	Putative GPI-anchored adhesin-like protein		2.22
CAALFM_C400650WA	HIS5	histidinol-phosphate transaminase		2.18
CAALFM_C302790WA	-	hypothetical protein		2.16
CAALFM_C703310WA	-	hypothetical protein		2.15
CAALFM_CR06770CA	-	hypothetical protein		2.12
CAALFM_C400080CA	-	hypothetical protein		2.11
CAALFM_CR06460WA	GST3	Glutathione S-transferase		2.07
CAALFM_C106860WA	-	hypothetical protein		2.06
CAALFM_C111320CA	-	hypothetical protein		2.06
CAALFM_CR00200WA	PCK1	phosphoenolpyruvate carboxykinase		2.06
CAALFM_C106550WA	GLT1	glutamate synthase (NADH)		2.02

CAALFM_C204460WA	LYS22	Homocitrate synthase, minor isoform		2.01
CAALFM_C100170WA	LEU4	2-isopropylmalate synthase		2.00
CAALFM_C401800WA	-	hypothetical protein		1.99
CAALFM_C601430CA	FRP6	Putative ammonia transport protein		1.97
CAALFM_C202030WA	ARO3	3-deoxy-7-phosphoheptulonate synthase		1.93
CAALFM_C102620CA	HOM6	homoserine dehydrogenase		1.84
CAALFM_CR01930CA	BIO2	biotin synthase		1.84
CAALFM_C306920WA	-	putative phosphomutase		1.83
CAALFM_C109580CA	LIP1	Secreted lipase		1.82
CAALFM_C503130WA	SUT1	Zn2Cys6 transcription factor involved in sterol uptake		1.82
CAALFM_C301060WA	-	hypothetical protein		1.80
CAALFM_C301480CA	RKI1	ribose-5-phosphate isomerase		1.78
CAALFM_C402150CA	-	hypothetical protein		1.77
CAALFM_C401200CA	AAT22	Aspartate aminotransferase		1.76
CAALFM_C305580CA	GAP2	amino acid permease		1.76
CAALFM_C601500CA	TRY5	Zn(II)2Cys6 transcription factor		1.75
CAALFM_C405320WA	LYS1	saccharopine dehydrogenase (NAD+%2C L-lysine-forming)		1.74
CAALFM_C206990WA	-	hypothetical protein		1.74
CAALFM_C603320WA	-	hypothetical protein		1.73
CAALFM_C102820WA	LYS2	L-aminoadipate-semialdehyde dehydrogenase		1.73
CAALFM_C205360CA	BIO32	Putative class III aminotransferase with a predicted role in biotin biosynthesis		1.72
CAALFM_C202550CA	-	hypothetical protein		1.72
CAALFM_C108410CA	SAM4	S-adenosylmethionine-homocysteine S-methyltransferase		1.72
CAALFM_CR07740WA	-	hypothetical protein		1.68

CAALFM_C102270CA	-	hypothetical protein		1.68
CAALFM_C108980CA	ZWF1	glucose-6-phosphate dehydrogenase		1.68
CAALFM_C300650WA	NGT1	N-acetylglucosamine (GlcNAc)-specific transporter		1.67
CAALFM_C404160WA	-	hypothetical protein		1.67
CAALFM_C107200WA	YMC1	organic acid transporter		1.66
CAALFM_CR05480WA	-	mitochondrial 2-oxodicarboxylate carrier		1.65
CAALFM_C503060CA	TNA1	Putative nicotinic acid transporter		1.65
CAALFM_C402230CA	-	hypothetical protein		1.64
CAALFM_C103720CA	-	hypothetical protein		1.64
CAALFM_CR00640WA	ACC1	acetyl-CoA carboxylase		1.64
CAALFM_C401340WA	-	hypothetical protein		1.64
CAALFM_C205990CA	-	hypothetical protein		1.63
CAALFM_CR10570CA	YHB4	Protein related to flavohemoglobins		1.62
CAALFM_C204280WA	-	hypothetical protein		1.61
CAALFM_C306860CA	-	aldo-keto reductase superfamily protein		1.61
CAALFM_C201010WA	HGT8	High-affinity glucose transporter of the major facilitator superfamily		1.61
CAALFM_C104020CA	CSH1	Aldo-keto reductase		1.60
CAALFM_C304270CA	CTP1	Putative citrate transport protein		1.59
CAALFM_C101620CA	-	hypothetical protein		1.58
CAALFM_C108900WA	-	hypothetical protein		1.58
CAALFM_C304830CA	FAS2	trifunctional fatty acid synthase subunit		1.58
CAALFM_C205890CA	IDP1	isocitrate dehydrogenase (NADP(+))		1.58
CAALFM_C305220WA	CDR1	ATP-binding cassette multidrug transporter		1.57
CAALFM_C604310WA	PEX11	Putative peroxisomal membrane protein		1.57
CAALFM_C200510WA	-	hypothetical protein		1.57

CAALFM_C400830WA	SPS20	Peroxisomal 2,4-dienoyl-CoA reductase		1.56
CAALFM_C602500CA	GCV1	glycine decarboxylase subunit T		1.55
CAALFM_CR06340CA	PGI1	glucose-6-phosphate isomerase		1.54
CAALFM_C305210CA	DYN1	dynein heavy chain		1.54
CAALFM_C105950CA	-	hypothetical protein		1.54
CAALFM_C206100WA	PMT4	dolichyl-phosphate-mannose-protein mannosyltransferase		1.54
CAALFM_CR06810WA	HHT2	histone		1.53
CAALFM_CR01400WA	LYS12	homoisocitrate dehydrogenase		1.53
CAALFM_C302750WA	-	hypothetical protein		1.52
CAALFM_C101360CA	-	glycogenin glucosyltransferase		1.51
CAALFM_CR07760WA	-	mitochondrial 37S ribosomal protein RSM19		-1.50
CAALFM_C501830CA	HAL9	Putative Zn(II)2Cys6 transcription factor		-1.50
CAALFM_CR05170CA	FDH1	formate dehydrogenase (NAD+)		-1.51
CAALFM_C700940WA	-	serine-type carboxypeptidase		-1.51
CAALFM_C701680CA	-	hypothetical protein		-1.52
CAALFM_CR08420WA	-	hypothetical protein		-1.53
CAALFM_C106790CA	MRPL19	mitochondrial 54S ribosomal protein YmL19		-1.53
CAALFM_C304530CA	TEC1	TEA/ATTS transcription factor		-1.54
CAALFM_C101990WA	HSP30	Putative heat shock protein		-1.54
CAALFM_C303690WA	-	hypothetical protein		-1.55
CAALFM_C504620CA	-	membrane insertase		-1.55
CAALFM_CR04820WA	-	hypothetical protein		-1.55
CAALFM_CR03110WA	-	glutamate--tRNA ligase		-1.55
CAALFM_C501700WA	MRPL36	mitochondrial 54S ribosomal protein YmL36		-1.55
CAALFM_C402740WA	-	hypothetical protein		-1.56

CAALFM_CR02470WA	-	uncharacterized ncRNA		-1.56
CAALFM_C402130WA	CYT2	cytochrome c1 heme lyase		-1.56
CAALFM_C104450CA	FMA1	Putative oxidoreductase		-1.56
CAALFM_C403820CA	HRQ2	Protein of unknown function		-1.57
CAALFM_C307890WA	TIM8	protein transporter		-1.57
CAALFM_C300880WA	KGD1	alpha-ketoglutarate dehydrogenase		-1.57
CAALFM_CR07170WA	-	hypothetical protein		-1.60
CAALFM_C300030CA	-	hypothetical protein		-1.61
CAALFM_C300010CA	-	hypothetical protein		-1.61
CAALFM_C700510WA	MAC1	Copper fist transcription factor		-1.62
CAALFM_CR00620CA	ARG1	argininosuccinate synthase		-1.62
CAALFM_CR07320CA	-	mitochondrial 54S ribosomal protein YmL4		-1.62
CAALFM_C104300CA	SSA2	Hsp70 family chaperone		-1.62
CAALFM_C104290CA	ACS2	acetate--CoA ligase		-1.63
CAALFM_C400520WA	PAM18	Predicted component of the presequence translocase-associated import motor (PAM complex) involved in protein import into mitochondrial matrix		-1.63
CAALFM_C207680WA	-	mitochondrial 54S ribosomal protein YmL7/YmL5		-1.63
CAALFM_C302270WA	-	hypothetical protein		-1.63
CAALFM_C105470WA	MTM1	Ortholog(s) have pyridoxal phosphate binding activity		-1.64
CAALFM_C102880CA	MIA40	Predicted component of the mitochondrial intermembrane space import machinery		-1.64
CAALFM_CR04340WA	PIM1	ATP-dependent Lon protease		-1.65
CAALFM_C403890WA	PTP2	tyrosine protein phosphatase		-1.66
CAALFM_C405020WA	IMG2	mitochondrial 54S ribosomal protein IMG2		-1.66

CAALFM_C108970WA	-	uncharacterized ncRNA		-1.67
CAALFM_C209350WA	TAZ1	lysophosphatidylcholine acyltransferase		-1.68
CAALFM_C501640WA	MAS2	mitochondrial-processing protease subunit alpha		-1.68
CAALFM_C400820WA	-	hypothetical protein		-1.69
CAALFM_C503510CA	-	hypothetical protein		-1.69
CAALFM_C303190CA	-	hypothetical protein		-1.70
CAALFM_C101740WA	CTN1	carnitine O-acetyltransferase		-1.70
CAALFM_C206460WA	RTA3	7-transmembrane receptor protein involved in regulation of asymmetric lipid distribution in plasma membrane		-1.70
CAALFM_C601360WA	-	hypothetical protein		-1.71
CAALFM_C703930CA	MAM33	Putative mitochondrial acidic matrix protein		-1.72
CAALFM_C300360WA	-	hypothetical protein		-1.74
CAALFM_C504530WA	-	mitochondrial 37S ribosomal protein RSM26		-1.74
CAALFM_C110360CA	-	hypothetical protein		-1.74
CAALFM_C112990WA	-	uncharacterized ncRNA		-1.75
CAALFM_C105510CA	RPS27A	ribosomal 40S subunit protein S27A		-1.77
CAALFM_C113000WA	-	uncharacterized ncRNA		-1.77
CAALFM_C500350CA	-	hypothetical protein		-1.78
CAALFM_C102730WA	-	hypothetical protein		-1.79
CAALFM_C201180WA	COX17	copper metallochaperone		-1.79
CAALFM_C603030WA	SAP5	Biofilm-specific aspartyl protease		-1.82
CAALFM_C108960WA	-	uncharacterized ncRNA		-1.83
CAALFM_CR08880CA	-	hypothetical protein		-1.87
CAALFM_C203390CA	HSP78	chaperone ATPase		-1.89
CAALFM_C104820CA	-	putative mitochondrial 54S ribosomal protein RTC6		-1.90

CAALFM_C603230WA	ARG3	ornithine carbamoyltransferase		-1.91
CAALFM_C112240CA	-	putative alanine--tRNA ligase		-1.91
CAALFM_C101750WA	-	hypothetical protein		-1.91
CAALFM_C100980WA	-	hypothetical protein		-1.92
CAALFM_C105270CA	-	hypothetical protein		-1.92
CAALFM_C112220WA	CDC6	AAA family ATPase		-1.93
CAALFM_C402770CA	-	cell proliferation protein		-1.94
CAALFM_C503410CA	-	mitochondrial 54S ribosomal protein YmL20		-1.95
CAALFM_C101730WA	tA(AGC)3	tRNA-Ala		-1.95
CAALFM_C703000CA	-	mitochondrial 54S ribosomal protein YmL49		-1.97
CAALFM_C602210WA	-	hypothetical protein		-2.00
CAALFM_C500280CA	-	ribonuclease		-2.04
CAALFM_C601990WA	PLB1	Phospholipase B	-4.03±0.13	-2.13
CAALFM_C403470CA	ECE1	hypha-specific protein	-2.52±0.17	-2.15
CAALFM_C107530WA	-	hypothetical protein		-2.15
CAALFM_C201700CA	HAP42	Predicted transcription factor	-2.48±0.25	-2.15
CAALFM_C503770CA	-	hypothetical protein		-2.19
CAALFM_C303270WA	-	hypothetical protein		-2.20
CAALFM_C407140WA	-	hypothetical protein		-2.20
CAALFM_C200760CA	-	hypothetical protein		-2.22
CAALFM_C114090WA	-	hypothetical protein		-2.22
CAALFM_CR03870WA	-	hypothetical protein		-2.23
CAALFM_CR06950CA	ATX1	copper metallochaperone	-2.57±0.34	-2.26
CAALFM_C203950WA	-	mitochondrial 54S ribosomal protein YmL24/YmL14		-2.27
CAALFM_C208890WA	-	hypothetical protein		-2.27

CAALFM_C301340WA	-	hypothetical protein		-2.29
CAALFM_CR06100CA	-	hypothetical protein		-2.30
CAALFM_C600920WA	-	hypothetical protein		-2.31
CAALFM_C403570WA	HWP1	Hyphal cell wall protein		-2.31
CAALFM_C110170WA	-	hypothetical protein		-2.32
CAALFM_CR06490CA	HSP60	chaperone ATPase	-1.34±0.47	-2.35
CAALFM_CR07540CA	-	hypothetical protein		-2.36
CAALFM_C112080WA	LCB4	sphinganine kinase		-2.41
CAALFM_C102070WA	HSP31	Putative 30 kda heat shock protein	-2.38±0.09	-2.41
CAALFM_C402070WA	PHO114	Acid phosphatase		-2.43
CAALFM_CR08830WA	-	hypothetical protein		-2.49
CAALFM_C106370CA	PBR1	Protein of unknown function, required for cohesion, adhesion, and biofilm formation	-5.43±1.29	-2.49
CAALFM_C305080WA	-	hypothetical protein		-2.52
CAALFM_C501840CA	TAC1	Zn(2)-Cys(6) transcriptional activator of drug-responsive genes	-2.18±0.28	-2.59
CAALFM_C204010CA	HSP21	Small heat shock protein	-2.42±0.16	-2.63
CAALFM_C204470WA	ADH3	Putative NAD-dependent (R,R)-butanediol dehydrogenase	-1.57±0.06	-2.64
CAALFM_C503600WA	MDJ1	Putative member of the HSP40 (DnaJ) family of chaperones	-2.77±0.38	-2.68
CAALFM_C210070WA	-	hypothetical protein		-2.70
CAALFM_C204000CA	-	hypothetical protein		-2.70
CAALFM_C113100WA	-	hypothetical protein	-2.18±0.43	-2.89
CAALFM_C207380WA	SSC1	Hsp70 family ATPase	-2.99±0.47	-3.00
CAALFM_C203110WA	-	hypothetical protein	-5.35±1.16	-3.01
CAALFM_C401910WA	-	transcription factor TFIIIC subunit		-3.19
CAALFM_CR03120WA	-	hypothetical protein		-3.19

CAALFM_C401900CA	MDH1-1	malate dehydrogenase	-3.93±0.37	-4.05
CAALFM_C105260CA	SDH1	succinate dehydrogenase flavoprotein subunit		-4.28
CAALFM_C108790WA	TPO3	spermine transporter	-3.36±0.19	-4.83

Table S3. PCR primers used in this study

Primer	Sequence (5'-3')
GSP1-F[a]	TGAAGTCCATCCATTAGGAT
GSP1-R	ATCTCTATGCCAGTTTGGAA
OP4-F	TGCCGGTAGCTGGATTAAAG
OP4-R	CAGTGGCAGTAGAAGCAGTAG
SCW4-F	GGTGGTAGTGATGGTGGTAAAG
SCW4-R	GATCCTGAACCAGAGCCTAAAC
AGA1-F	CACCAGCTACTCCAATGACTAC
AGA1-R	CGGCAGCATAAGCATCATTTAC
PXP2-F	TTTGGAAGGTAGAGTTGGTATGG
PXP2-R	GCACTGGCAAATTGTCTTCTAC
PRY1-F	CTGACTCTGGTTCTGGCTTAC
PRY1-R	GAAGTTGAGGAGGAACCGATATT
PLB1-F	CGTGTCTGCTTACAATCCAAATC
PLB1-R	CATCTTCTCCACCGTCAACTAA
ECE1-F	GCTGGTATCATTGCTGATAT
ECE1-R	TTCGATGGATTGTTGAACAC
HAP42-F	CCCAATACCACAAGCAATGAAG
HAP42-R	CGACCACCACCACAACTATTA
ATX1-F	TGTTCAGGAGCCATTGAAAGAG
ATX1-R	GCTGAGTAGTTATAACATCAACAGTTTG
HSP31-F	ACACTGCTACCAAGGTCAATC
HSP31-R	CGGAGAATGCAGCCCATAA
PBR1-F	TCCGATAGTGAAGAAGTCAATGG
PBR1-R	GTAGCACCTGGAGTAACGTAAT
TAC1-F	CAGGGAATGACCCTAACGATAC

TAC1-R	CCCACATGGATAAGACCCATT
ADH3-F	GTCCAAGGATGTGGTGGATTAG
ADH3-R	ACCGCACCTTTAACATCAGAA
SSC1-F	GTTATCCTTGTCGGTGGTATGT
SSC1-R	AACAGCTTCATCAGGGTTGATA
MDJ1-F	CCAAGGTGCTGGTGTTACTAT
MDJ1-R	TGCAGGGATTTCAACAGTCTTA
TPO3-F	CTGGAGGACTTTCCACTGTTAAT
TPO3-R	GGACCAACACAGAACCCAATA
HWP1-F	TGGTGCTATTACTATTCCGG
HWP1-R	CAATAATAGCAGCACCGAAG
ALS3-F	CTAATGCTGCTACGTATAATT
ALS3-R	CCTGAAATTGACATGTAGCA
TEC1-F	AGGTTCCCTGGTTTAAGTG
TEC1-R	ACTGGTATGTGTGGGTGAT
RBT5-F	GCCAGAATGTGCCAAAGAATG
RBT5-R	CACCAGCAAATTGAGGCATAAC
IFD6-F	CCGTGAAGATGACAGAGAGTTG
IFD6-R	GACACAAGACACCACCACTATT
CSH1-F	AGTCAAAGACGACGCAGAAG
CSH1-R	TTCAGCTGCGGCTAAGATATG
HSP90-F	AGACACCACCATGTCTTCTTAC
HSP90-R	TCTTCAGCTCCATCGGTTTC

Figure S1. ESIMS spectrum of compounds 1 and 2.

(a) ESIMS spectrum of compound 1

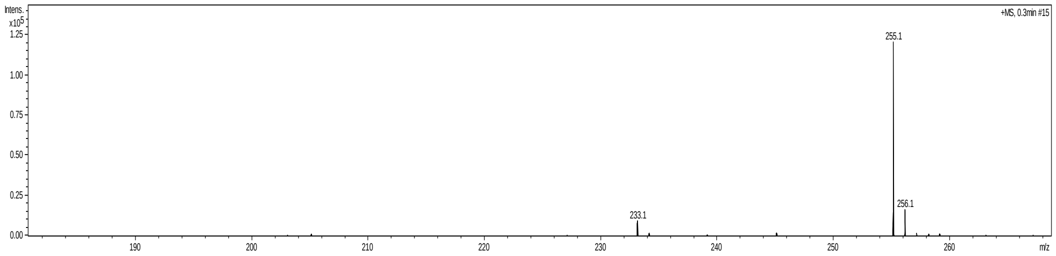

(b) ESIMS spectrum of compound 2

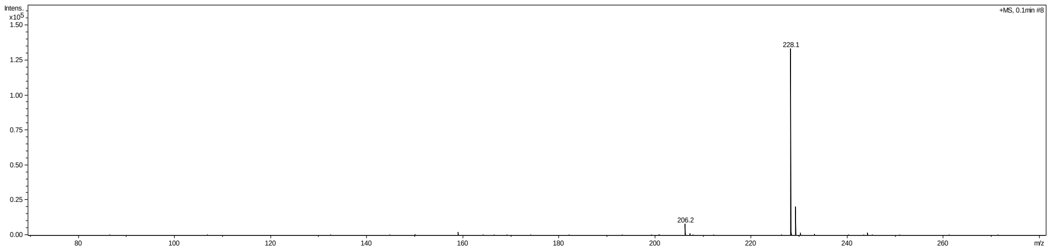

Figure S2. Growth curves of *C. albicans* cells treated by compound **1** (50 μg/mL) and compound **2** (100 μg/mL).

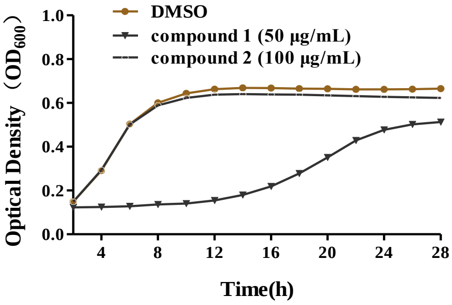

Supplement: Supplementary file 1 [file marinedrugs-17-00442-s001.pdf]
